# A Direction-of-Arrival Estimation Algorithm Based on Compressed Sensing and Density-Based Spatial Clustering and Its Application in Signal Processing of MEMS Vector Hydrophone

**DOI:** 10.3390/s21062191

**Published:** 2021-03-21

**Authors:** Huichao Yan, Ting Chen, Peng Wang, Linmei Zhang, Rong Cheng, Yanping Bai

**Affiliations:** 1 School of Information and Communication Engineering, North University of China, Taiyuan 030051, China; b1705012@st.nuc.edu.cn; 2 Department of Mathematics, School of Science, North University of China, Taiyuan 030051, China; s1808003@st.nuc.edu.cn (T.C.); wpmath@nuc.edu.cn (P.W.); s1708029@st.nuc.edu.cn (L.Z.); chengro@nuc.edu.cn (R.C.)

**Keywords:** direction of arrival estimation, compressed sensing, density-based spatial clustering, MEMS vector hydrophone, small snapshots, low signal to noise ratio

## Abstract

Direction of arrival (DOA) estimation has always been a hot topic for researchers. The complex and changeable environment makes it very challenging to estimate the DOA in a small snapshot and strong noise environment. The direction-of-arrival estimation method based on compressed sensing (CS) is a new method proposed in recent years. It has received widespread attention because it can realize the direction-of-arrival estimation under small snapshots. However, this method will cause serious distortion in a strong noise environment. To solve this problem, this paper proposes a DOA estimation algorithm based on the principle of CS and density-based spatial clustering (DBSCAN). First of all, in order to make the estimation accuracy higher, this paper selects a signal reconstruction strategy based on the basis pursuit de-noising (BPDN). In response to the challenge of the selection of regularization parameters in this strategy, the power spectrum entropy is proposed to characterize the noise intensity of the signal, so as to provide reasonable suggestions for the selection of regularization parameters; Then, this paper finds out that the DOA estimation based on the principle of CS will get a denser estimation near the real angle under the condition of small snapshots through analysis, so it is proposed to use a DBSCAN method to process the above data to obtain the final DOA estimate; Finally, calculate the cluster center value of each cluster, the number of clusters is the number of signal sources, and the cluster center value is the final DOA estimate. The proposed method is applied to the simulation experiment and the micro electro mechanical system (MEMS) vector hydrophone lake test experiment, and they are proved that the proposed method can obtain good results of DOA estimation under the conditions of small snapshots and low signal-to-noise ratio (SNR).

## 1. Introduction

Direction of arrival (DOA) estimation has been an important research field for scientific researchers because of its wide application scenarios. Its application in 5G and internet of things communication [1,2], medical imaging [3], earthquake monitoring [4] and underwater acoustic signal processing [5,6] greatly facilitates people’s production and life. With the rapid development of information technology, more and more application scenarios and complex application environment bring more challenges to DOA estimation technology. In real life, the signals are often mixed with noise and the effective signals are limited, so it is necessary to study the DOA estimation algorithm under the condition of small snapshot and low signal-to-noise ratio (SNR).

The early direction-finding algorithm is mainly developed around beamforming technology, which can enhance the desired signal and suppress interference by weighting each element in spatial filtering. However, the technology cannot distinguish the space target within a beam width, which limits the estimation accuracy of the technology. In order to improve the resolution of this technology and make the algorithm adapt to the changes of signal environment, researchers use various criteria to determine the weights [7]. The representative article is Capon’s minimum variance method [8]. However, this kind of method assumes that the signal is a spatially stationary random process, and this assumption is not valid in real signals, the algorithm has a large error or even failure in low SNR environment, which leads to some limitations in application.

The emergence of subspace algorithms greatly improves the resolution of DOA estimation. The most classic method is multiple signal classification algorithm (MUSIC) [9], which uses the orthogonal characteristics of signal subspace and noise subspace to construct spatial spectral peaks, and then achieves super-resolution DOA estimation. Another classical algorithm uses the rotation invariance of signal subspace to achieve super-resolution estimation [10]. The above method and the improved method [11,12,13,14] depend on the statistical characteristics of the data, so it needs a lot of snapshots data to achieve accurate estimation. In addition, the above method cannot effectively distinguish the incident direction of coherent signal. The maximum likelihood based DOA estimation algorithm is a typical representative of subspace fitting algorithms, but it needs multidimensional search to solve the optimal solution, and the amount of computation is huge.

Sparse representation of signals is a signal processing technology that has attracted much attention in recent years. It shows the information contained in the signal as comprehensively as possible with a simple mathematical model, which makes the signal processing more convenient [15]. DOA estimation algorithm based on compressed sensing (CS-DOA) is proposed in recent years to achieve high-resolution estimation using signal sparsity [16]. Because it can realize DOA estimation for single snapshot data and coherent signals, this algorithm has been widely concerned. At present, CS-DOA is mainly divided into two strategies according to different signal reconstruction algorithms, one of which is greedy algorithm and its improvement algorithms [17,18,19]. This kind of method search for the support set of unknown signals iteratively, that is, the position of non-zero elements. However, when the wrong target is selected in the process, the target will be retained until the end, making the result biased. Another kind of strategy is based on convex optimization method, including basis pursuit de-noising (BPDN), Least absolute shrinkage and selection operator (LASSO) and $l_{1}-SVD$ algorithm [20,21,22]. This kind of algorithms needs to choose appropriate regularization parameters to ensure the performance of the algorithm. In addition, because the DOA estimation algorithm based on CS principle needs to divide the spatial domain into grids in advance. The angle of the real signal may not be within the dividing grid, leading to the bias of the result. A grid evolution DOA (GEDOA) estimation method is proposed in literature [23]. Literature [24] solves the mesh mismatch problem by introducing bias parameters into the signal model. In the process of grid fission, sparse Bayesian learning method is used to correct the position of spatial spectrum, so as to achieve fast and accurate DOA estimation. These above algorithms based on CS cannot guarantee the estimation performance at low SNR. There are also many literatures that focus on the research on the principle of CS in the mutual-prime array and the location of far and near field signals, and many fruitful works have been done in the above research area [25,26,27,28,29].

For the DOA estimation algorithms under the condition of small snapshot number, researchers have also explored other methods [30,31]. In reference [30], a Toeplitz matrix, using which to obtain the signal subspace and noise subspace, is constructed using any row of the received signal’s covariance matrix. Then, the DOA estimation of single snapshot signal is realized by using the subspace method. However, this method needs to know the number of signal sources, and it is still unable to achieve the correct estimation under the condition of low SNR.

Based on the above analysis, the DOA estimation is difficult under the condition of small snapshot number and low SNR. Therefore, this paper proposes a DOA estimation algorithm based on CS and density-based spatial clustering (DBSCAN). Firstly, calculate the power spectrum entropy of the signal, and select the appropriate regularization parameters; Secondly, the CS-DOA method based on the basis pursuit noise reduction strategy is used to estimate the DOA of the small snapshot data of the signal, and the peak data of the estimation result is saved; Finally, the saved peak data is clustered by DBSCAN, and calculate the cluster center value of each cluster, the number of clusters is the number of the signal sources and the cluster center value is the estimated value of DOA. Under the same conditions of low SNR and small snapshots, the proposed method can get effective DOA estimation, which has more advantages than the traditional super-resolution algorithms. In the real underwater acoustic experiment environment, when there is a short pulse signal, the DOA estimation still can be obtained by this method. The method proposed in this paper does not need to predict the number of signal sources. In the case of low SNR, small snapshots, coherent signals and a small number of abnormal points, it can still obtain effective DOA estimation, which has more advantages than traditional DOA methods.

The rest of this paper is arranged as follows: in the second section, the principle of DOA estimation based on CS and its characteristic analysis are introduced, as well as the power spectrum entropy, regularization parameter selection method and the clustering principle based on density. In the third section, the specific steps of the proposed method are introduced. In the fourth and fifth sections, the effectiveness and advantages of the proposed method in simulation experiments and real underwater acoustic experiments of micro electro mechanical system (MEMS) vector hydrophone are verified, respectively. Finally, a conclusion of this paper is given in the sixth section.

## 2. Theoretical Basis and Analysis

### 2.1. Principles and Characteristics of DOA Estimation Based on CS Theory

#### 2.1.1. DOA Estimation Principle Based on CS Theory

CS theory is a set of new theory about sparse signal acquisition and compression which was put forward in 2006. It uses the characteristics of sparse signal to sample the signal and compress the data properly, which can effectively reduce the burden of data transmission and processing. It has attracted the attention of the majority of scientific researchers and has been widely used in various fields of signal processing. Now it has formed a set of perfect theoretical basis [32]. The DOA estimation based on CS theory exactly uses the sparsity of the signal to process the signal.

Suppose that there are K far-field narrow-band signals incident on a uniform linear array composed of M antennas, and the incident angle of the k−th signal is $\theta_{k}$. The single snapshot data vector received by the array at time t can be expressed as

(1) x(t)=A(θ)s(t)+n(t)

where, $s(t)={\left[s_{1},s_{2},\cdots ,s_{K}\right]}^{T}$ represents K source vectors; $A(\theta )=[a(\theta_{1}),a(\theta_{2}),\cdots ,a(\theta_{K})]$ is the direction matrix; n(t) is receiving noise for array.

**Figure 1:**
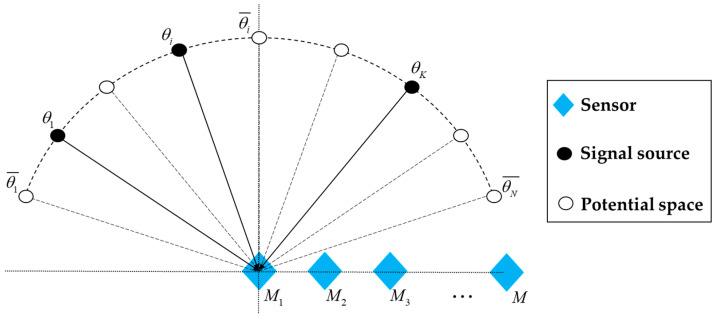
Sparse representation of array signals.

The potential incidence angles of space are divided, and the divided space angles are expressed as $\left\{\bar{\theta_{1}},\bar{\theta_{2}},\cdots ,\bar{\theta_{N}}\right\}$, As shown in Figure 1, a solid circle represents an actual incidence angle of space, and the hollow circle represents the incidence angle of potential space that does not exist. When the number of potential incidence angles N is much larger than the number of actual incidence angles, namely N≫K, the incident signal can be regarded as a sparse signal in the spatial domain.

In order to take advantage of the sparsity of the signal, the array manifold matrix A is extended to form a super complete redundant dictionary G, which contains all possible angles, namely

(2) $$G=[a(\theta_{1}),a(\theta_{2}),\cdots ,a(\theta_{i}),\cdots ,a(\theta_{N})],\;i=1,\cdots ,N$$

Combining Formulas (1) and (2), the Formula (1) can be transformed into a sparse representation problem, that is

(3) x=Gδ+n

where, δ is the N dimension coefficient vector. According to the sparse theory, the number of non-zero elements in δ in the dictionary is K, which means that the dictionary G is K-sparse. The angle of the vector corresponding to the position of the non-zero element represents the value of the incident angle θ, and the amplitude of the non-zero element is the amplitude of the signal at the sampling time.

Obviously, Formula (3) is an underdetermined equation, which cannot be solved directly. However, when the sensing matrix satisfies certain sparse reconstruction conditions, the signal can be reconstructed with a high probability. The sparser the signal is, the higher the reconstruction accuracy will be. Candes proved that the signal reconstruction can be solved by solving the minimum $l_{0}$ norm problem [33], which can be solved by the following equation

(4) $$\bar{\delta }=\mathrm{argmin}{\left\|\delta \right\|}_{0},\;s.t.x=G\delta +n$$

But $l_{0}-$norm minimum optimization algorithm is a non-deterministic polynomial hard problem. In order to ensure the sparsity of signal and the accuracy of signal reconstruction, Formula (4) is usually transformed into an optimization problem with inequality constraints

(5) $$\hat{\delta }=\mathrm{argmin}{\left\|\delta \right\|}_{1},\;s.t.{\left\|x-G\delta \right\|}_{2}^{2}\leq \gamma^{2}$$

where γ represents a possible noise standard deviation. In general, the solution of Formula (5) is transformed into the solution of the following unconstrained optimization problem

(6) $$\hat{\delta }=\underset{\delta }{\min}\lambda {\left\|\delta \right\|}_{1}+\frac{1}{2}{\left\|x-G\delta \right\|}_{2}^{2}$$

In the Formula (6), λ is a regularization parameter related to noise. In the process of signal reconstruction, it affects the sensitivity of DOA estimation to signal noise and largely affects the DOA estimation result is whether correct. The existing literature do not provide good suggestions for the selection of λ [34,35].

#### 2.1.2. Features of CS-DOA

In order to visually show the characteristics of the DOA estimation method based on the CS theory, and provide suggestions for the selection of the regularization parameter λ and the improvement of the method, this article designs some experiments for analysis, considering that different signal reconstruction methods have a great impact on the accuracy of DOA, the BPDN method has higher stability and accuracy than other reconstruction algorithms, therefore, this paper chooses BPDN method to reconstruct the signal, and uses the software for disciplined convex programming (CVX toolbox) to realize the reconstruction process [36].

**Figure 2:**
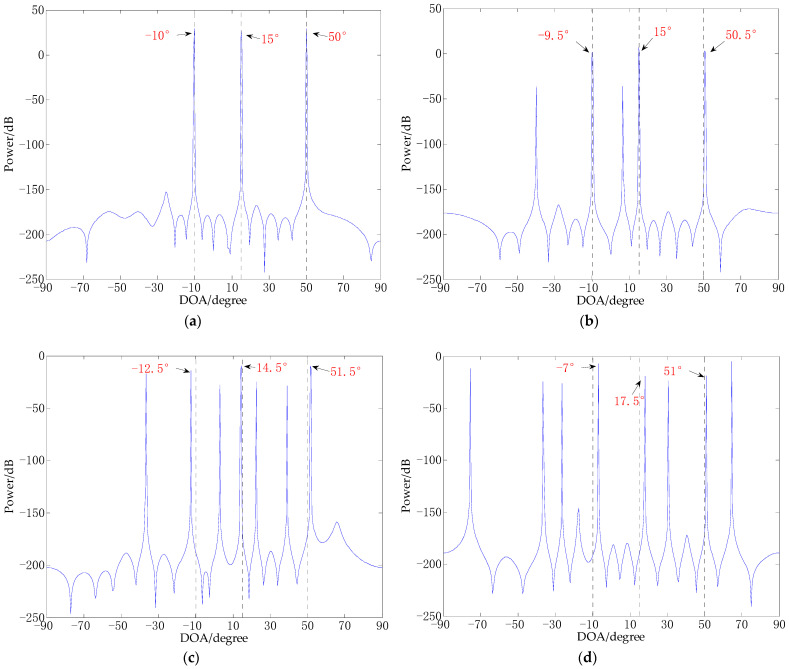
CS-DOA experiment under the same regularization parameter and different SNR. (**a**) SNR = 30 dB; (**b**) SNR = 10 dB; (**c**) SNR = −5 dB; (**d**) SNR = −10 dB.

Experiment 1: CS-DOA experiment under the same regularization parameter and different SNR. The number of array elements is 10, and the three incident angles are −10°, 15°, 50°. In this experiment, the regularization parameter is 10, random Gaussian white noises are added, the SNRs are 30 dB, 10 dB, −5 dB, −10 dB, respectively. The results of the experiments are shown in Figure 2.

It can be seen from Figure 2 that the lower the SNR, the falser peaks, and the spectral value of the false peaks becomes stronger. This indicates that the noises make CS-DOA unable to obtain the correct DOA estimation, and the lower the SNR, the greater the error of DOA estimation. Due to the existence of a large number of false peaks, the number of sources is also difficult to accurately estimate. Even though the algorithm allows some noises in the model, it is still difficult to obtain good DOA estimation results in a low SNR environment.

**Figure 3:**
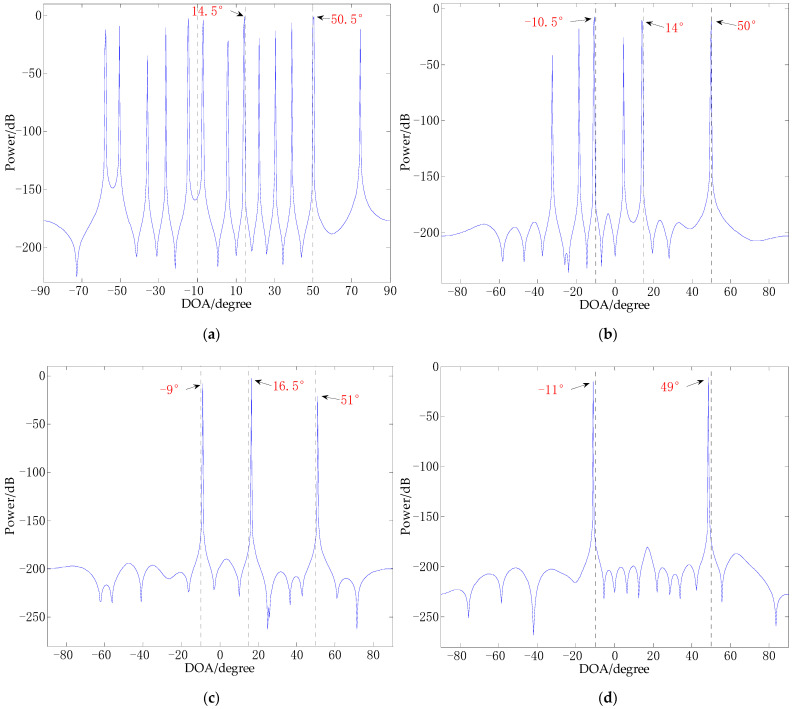
CS-DOA experiment with the same SNR and different regularization parameters. (**a**) λ=0.5; (**b**) λ=5; (**c**) λ=15; (**d**) λ=30.

Experiment 2, CS-DOA experiment with the same SNR and different regularization parameters. The number of array elements is 10, and the three incident angles are −10°, 15°, 50°. In this experiment, the SNR is 0dB, and the regularization parameters are 0.5, 5, 15, and 30 respectively. The results of the experiments are shown in Figure 3.

It can be seen from Figure 3 that under the same SNR environment, the size of the regularization parameter is related to the adaptability of the CS-DOA algorithm to noise. The larger the regularization parameter, the stronger the adaptability of the CS-DOA algorithm to noise. When the regularization parameter is large enough, the correct DOA estimate will even be filtered out, as shown in Figure 3d. It can be seen that the complexity of signal noise can provide a reference for the selection of regularization parameters. How to determine the noise complexity of the signal and provide suggestions for the selection of regularization parameters will be discussed in the next section.

In addition, combining Figure 2 and Figure 3, it can be seen that in experiments with different SNRs and regularization parameters, the CS-DOA algorithm can obtain the estimation result near the correct DOA value with a high probability. Even if there are some false peaks in the signal reconstruction process, and even the correct solution is lost, as the number of snapshots increases, if we can use the CS-DOA estimation results obtained from a small number of snapshots for comprehensive analysis, it seems that the correct number of sources and the correct DOA estimation value can be obtained. How to use these results for analysis will be further discussed in the following content.

### 2.2. Selection of Regularization Parameters

Combining the content of the previous section, we propose that the appropriate regularization parameters can be selected by determining the complexity of the signal noise. In fact, no specific signal component must be regarded as noise. Noise is just a part of the signal that we do not need in the process of signal processing. For example, the sound made by a ship engine when it is running is regarded as a kind of noise for tourists or underwater creatures. When we use a sound to judge the type, size and direction of the ship, the sound emitted by its engine is the useful signal we need, and other sounds become useless noise [37]. In reality, we only need part of the information in the signal for DOA estimation, and the signal is usually composed of various frequency components and distributions.

Power spectral entropy (PSE) is an extension of information entropy in the frequency domain. Because it can be used to describe the characteristics of frequency changes, it has been widely used in the analysis of the frequency complexity of various signals [38]. Generally, the smaller the power spectrum entropy of the signal, the stronger the signal sparsity. This paper selects the power spectrum entropy to represent the frequency complexity of the signal, which can indirectly reflect the magnitude of signal noise.

The calculation of power spectrum entropy includes the following three steps:

Step 1, Calculate the power spectrum of signal x(t):

(7) $$s(f)=\frac{1}{2\pi L}{\left|x(w)\right|}^{2}$$

where L is the signal length and x(w) is the Fourier transform of x(t);

Step 2, Calculate the probability density function of the spectrum of all frequency components by normalization:

(8) $$P_{i}=\frac{s(f_{i})}{\sum_{k=1}^{N}s(f_{k})},(i=1,2,3,\cdots ,N)$$

where $s(f_{i})$ is the spectral energy of frequency component $f_{i}$, $P_{i}$ is the corresponding probability density, and N is the number of frequency components in the fast Fourier transform of the total probability density;

Step 3, Calculate the value of PSE:

(9) $$H=-\sum_{k=1}^{N}P_{i}\log P_{i}$$

Different from the traditional spectrum analysis, power spectrum entropy quantifies the frequency complexity of the signal in its calculation process, which makes the analysis of the signal characteristics more convenient.

**Figure 4:**
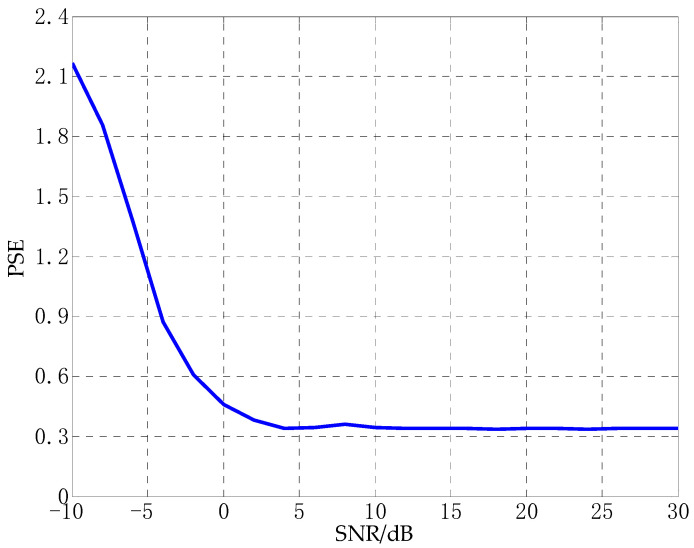
The trend of the power spectrum entropy of the signal under different noise environments.

Figure 4 shows the trend of power spectrum entropy of signals in different SNR environments obtained through 100 independent Monte Carlo tests. It can be seen from Figure 4 that the power spectral entropy of the signal decreases as the SNR increases, and eventually tends to a stable state. When the SNR is greater than 5 dB, the power spectrum entropy of the signal is between 0.3 and 0.4. When the SNR changes from 5 dB to −10 dB, the stronger the noise, the greater the power spectrum entropy of the signal.

**Figure 5:**
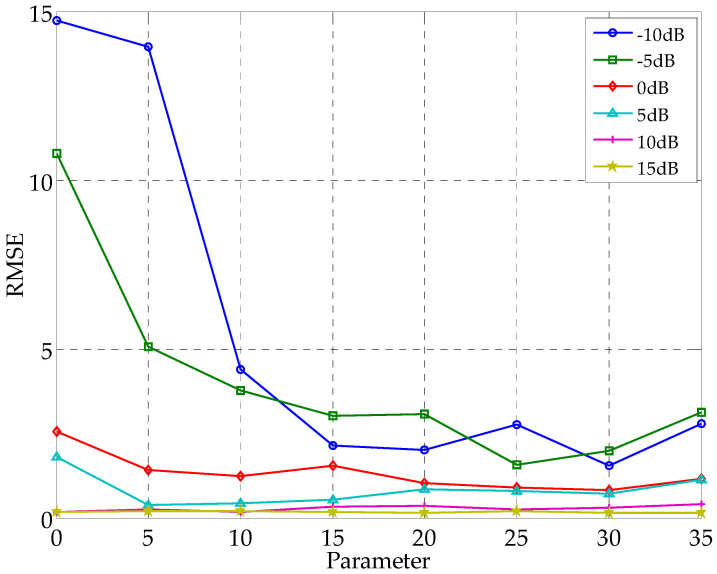
Root mean square error of CS-DOA estimation under different SNR and regularization parameters.

Through the analysis in Section 2.1.2. we know that when the regularization parameters are randomly selected, the signal reconstruction results may be affected by pseudo peaks. In order to analyze the influence of different regularization parameters on the orientation accuracy of the CS-DOA model, we repeated a large number of experiments for different regularization parameters under different noise intensity environments. Save the average value of the effective DOA estimates in the experiment, and find the root mean square error from the true angle. Figure 5 shows the root mean square error of the CS-DOA model with different regularization parameters in different noise environments.

It can be seen from Figure 5 that when the SNR is high, the DOA estimation error is small, and the selection of the regularization parameter has little effect on the DOA estimation result. In a low SNR environment, the smaller the regularization parameter, the greater the root mean square error of the DOA estimate. When the regularization parameter increases, the error gradually decreases. When the regularization parameter is greater than 30, the error has a rising trend. Combined with the analysis in Section 2.1.2, if the real signal energy is weak and the regularization parameter is too large, the correct DOA estimation may be lost.

Based on the above analysis, in the DOA estimation model introduced in this article, the regularization parameters are selected according to the noise complexity represented by the power spectrum entropy. When the power spectrum entropy value is about 0.3, the regularization parameter is selected between 5–10, and when the power spectrum entropy is large, the regularization parameter is selected between 10–20.

### 2.3. Density-Based Spatial Clustering

It can be seen from Section 2.1.2, that in experiments with different SNRs and regularization parameters, the CS-DOA algorithm can obtain the estimation results near the true angle with a high probability, and the false peaks caused by noises and other unstable factors are Scattered in different locations. When there are a small number of snapshots, the peaks data can be analyzed to determine where the CS-DOA estimation are denser, and the number of sources and the DOA estimation can be obtained. It is unrealistic to analyze large amounts of data manually. The clustering method seems to be a good choice. It can analyze data with similar attributes in the collection. General clustering methods usually need to input the number of clusters in advance. In reality, we cannot determine the number of classifications in advance, which may bring wrong results. In this article, we use DBSCAN to process the peaks data of CS-DOA.

DBSCAN characterizes the data according to the tightness of the data distribution [39]. It uses a set of parameters (ε,MinPts) called “neighborhood” to examine the connectivity between samples, and continuously expands clusters based on connectable samples to obtain the final clustering results.

In order to describe the DBSCAN algorithm in detail, the following definitions need to be understood:

ε−neighborhood: For $h_{j}\in H$, its ε−neighborhood contains samples in the sample set H whose distance from $h_{j}$ is less than or equal to ε. That is $N_{\varepsilon }(h_{j})=\left\{h_{i}\in H|dist(h_{i},h_{j})\leq \varepsilon \right\}$, where $H=\left\{h_{1},h_{2},\cdots ,h_{m}\right\}$ is the sample data set, and dist(·,·) represents the Euclidean distance;

Core object: If the ε−neighborhood of $h_{j}$ contains at least MinPts samples, that is, $\left|N_{\varepsilon }(h_{j})\right|\geq MinPts$, then $h_{j}$ is a core object;

Directly density-reachable: If $h_{j}$ is located in the ε−neighborhood of $h_{i}$, and $h_{i}$ is the core object, then $h_{j}$ is directly reachable by $h_{i}$ density;

Density reachable: For $h_{i}$ and $h_{j}$, if there is a sample sequence $q_{1},q_{2},\cdots ,q_{n}$, where $q_{1}=h_{i},q_{n}=h_{j}$, and $q_{i+1}$ is directly reached by the density of $q_{i}$, then $h_{j}$ is reachable by the density of $h_{i}$;

Density-connected: For $h_{i}$ and $h_{j}$, if there is $h_{k}$ so that both $h_{i}$ and $h_{j}$ are reachable by the density of $h_{k}$, then $h_{i}$ and $h_{j}$ are density connected.

**Figure 6:**
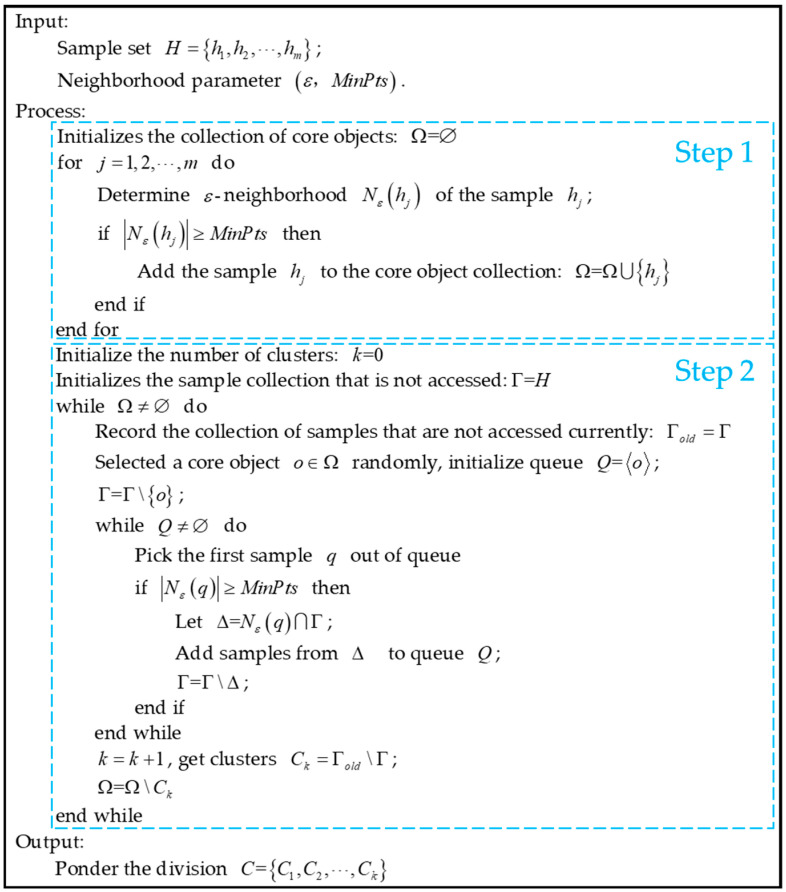
DBSCAN pseudo code flow chart.

DBSCAN is a collection of the largest density-connected samples derived from the above-mentioned density reachability relationship. The clustering process of DBSCAN can be roughly divided into two steps: Step 1. Find all the core objects according to the given neighborhood parameter (ε,MinPts); Step 2. Take any core object as the starting point to find the clusters generated by the samples whose density is reachable. Stop rule for all core objects are accessed. The pseudo-code flow of DBSCAN is shown in Figure 6.

Based on the above analysis, the sample set H is the DOA estimation result of the small snapshot data, and the neighborhood parameters can also be selected according to the characteristics of the signal data. The selection idea will be discussed in the following part.

## 3. The Method Proposed in This Paper (CS-DOA-DBSCAN)

Combined with the above basic theories and analysis, an estimation algorithm of the DOA based on CS theory and DBSCAN is proposed (CS-DOA-DBSCAN). The specific steps of the algorithm are as follows:

Step1: The power spectrum entropy of the signal is calculated, and then the regularization parameter range is determined;

Step2: Based on the principle of CS, the DOA of small snapshots data was estimated, and the first 15 maximum peaks data coordinates of each snapshot were saved for the further processing;

Step3: Cluster the peaks data saved in step 2 through DBSCAN to obtain the number of clusters C, which are the number of signal sources;

Step4: Calculate the cluster center value of each cluster, which is the final DOA estimation.

Here are the more details for this algorithm: Although step 1 cannot lock the regularization parameter into an accurate value, the regularization parameter can be locked within a relatively small value range. Selecting the regularization parameter within this range can ensure that there are relatively few false peaks in the subsequent estimation of the DOA, and the estimation results are relatively stable, so as not to produce large fluctuations.

Combined with the analysis in Section 2.1.2. In step 2, the peaks data obtained in the CS-DOA estimation of each snapshot is generally not too much, so the 15 maximum peaks data before each snapshot are retained. In general, DOA estimates near the true angles will be retained. If too much peaks are saved, the workload of the algorithm will increase and the efficiency of the algorithm will be reduced.

In step 3, the number of clusters C represents the actual number of signal sources, and the cluster center value represents the final DOA estimation. In general, we pay more attention to the information of incident angles. In order to facilitate clustering, we normalized the amplitude of the data saved in step 2. In the process of clustering, the choice of neighborhood parameter ε will affect the resolution of DOA estimation. If ε is too large, the DOA resolution will be reduced a lot, and noise will have more influence on the clustering results, leading to the deviation of DOA estimation. If ε is too small, in the case of low SNR, noise points may also be clustered into a DOA estimation, resulting in wrong estimation of the algorithm. In the experiment, the value of ε can be determined according to the complexity of the noise.

**Figure 7:**
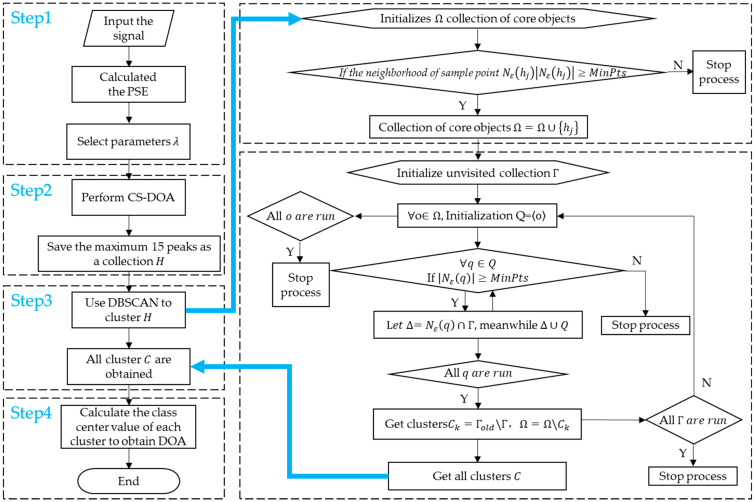
Flow chart of CS-DOA-DBSCAN algorithm.

MinPts represent the minimum number of samples contained in the ε−neighborhood. The selection of MinPts are related to the number of snapshots of the data. When the noise level is moderate or mild, CS-DOA is not easy to lose the effective solution. The value of MinPts can be equal to or slightly greater than the number of snapshots. It is recommended that no more 1.5 times than the number of snapshots. When the noise is large, CS-DOA may lose the effective solution. The value of MinPts can be equal to or slightly smaller than the number of snapshots. The CS-DOA-DBSCAN algorithm flow is shown in Figure 7.

## 4. Simulation

To verify the effectiveness of the proposed algorithm and its advantages in DOA estimation under the small snapshot and low SNR, several simulation experiments were designed to verify the performance of the proposed algorithm, which was also compared with the traditional super-resolution algorithms.

### 4.1. DOA Experimental Performance Analysis of a Small Number of Signal Sources

**Figure 8:**
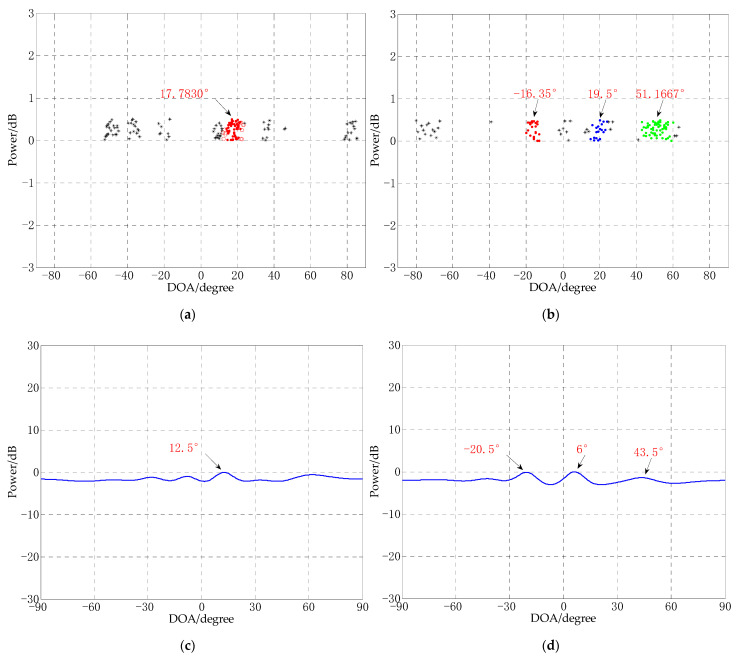

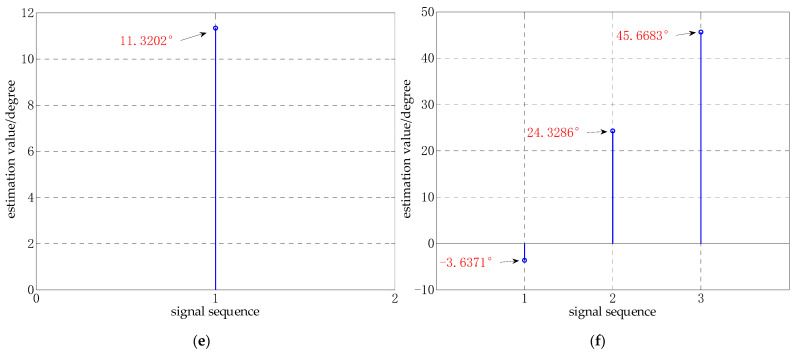
DOA estimation experiments for different algorithms (snapshots = 10, SNR = −10 dB). (**a**) CS-DOA-DBSCAN for single source signal, (**b**) CS-DOA-DBSCAN for multi-source signal, (**c**) MUSIC for single source signal, (**d**) MUSIC for multi-source signal, (**e**) TLS-ESPRIT for single source signal, (**f**) TLS-ESPRIT for multi-source signal.

In this experiment, the performance for single source and multi-source signals of the proposed algorithm was tested under the conditions of small snapshots and low SNR, and also compared with MUSIC algorithm and TLS-ESPRIT algorithm under the same conditions. The number of array elements is 8, the distance between array elements is 0.5 m, the number of snapshots is 10, the SNR is −10 dB, the incident angle of the single source signal is 17°, and the three incident angles of the multi-source signal are −15°, 20° and 50°, respectively. The experimental results are shown in Figure 8.

It can be seen in Figure 8a,b that under the conditions of small snapshots and low SNR, CS-DOA-DBSCAN can obtain DOA estimation effectively, and regardless of single source or multi-sources signals, the estimation errors are small. While the MUSIC algorithm cannot obtain DOA estimation effectively under the same conditions. It can be seen from the position of the extreme points marked in Figure 8c,d that the maximum extreme points obtained by the algorithm corresponding to the number of sources are not the correct directions of arrival. It can be seen from Figure 8e,f that the TLS-ESPRIT algorithm also cannot correctly estimate the directions of arrival.

**Figure 9:**
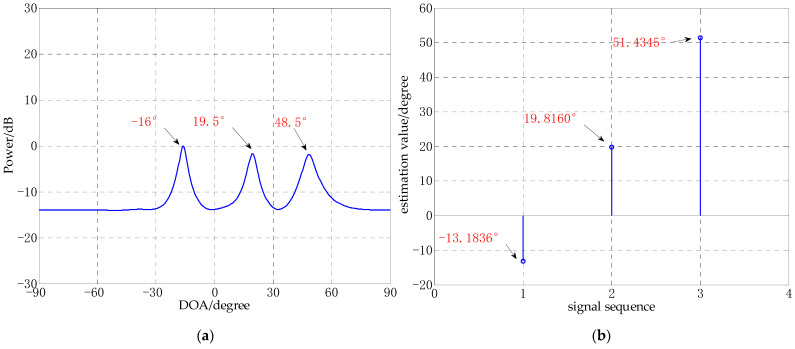
DOA estimation of MUSIC and TLS-ESPRIT (snapshots = 1000, SNR = −10 dB). (**a**) MUSIC, (**b**) TLS-ESPRIT.

To further illustrate the advantages of CS-DOA-DBSCAN in DOA estimation under the condition of small snapshots and low SNR, Figure 9 shows the DOA estimation results of the multi-sources signals under the condition of 1000 snapshots for the MUSIC algorithm and the TLS-ESPRIT algorithm based on the above experiments.

It can be seen from Figure 9 that when the number of snapshots increases to 1000, both MUSIC and TLS-ESPRIT can obtain effective DOA estimates. However, it needs to know the number of signal sources in advance. We know, the high-quality sampling is difficult in the realistic environment. The proposed algorithm does not need to know the number of sources in advance, and it can automatically cluster the number of sources through density clustering. Even if CS-DOA loses a correct DOA estimation in a small part of snapshots, it will not affect the clustering results of small snapshots data.

### 4.2. DOA Experimental Performance Analysis of Multiple Signal Sources

**Figure 10:**
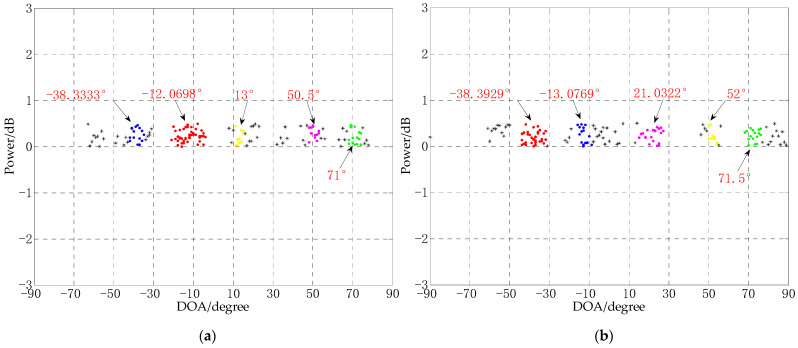
The DOA estimation experiment of the CS-DOA-DBSCAN algorithm in a multi-source environment, (**a**) the number of array elements is 6; (**b**) the number of array elements is 8.

In order to verify the DOA estimation performance of the algorithm proposed in this paper when there are a large number of signal sources, the following two experiments are designed: the number of array elements are 6 and 8, the other experimental environments are the same, the distance between the array elements is 0.5 m, the number of snapshots are 10, the SNR is −10 dB, five signal sources are −40°, −15°, 20°, 50° and 70° respectively, and the experimental results are shown in Figure 10.

It can be seen from Figure 10 that both experiments can get the correct number of sources, but in the experiment where the number of array elements is 6, one of the angles has a large deviation, the true angle is 20°, and the estimated angle is 13°. In the experiment where the number of array elements is 8, the estimation error of each angle is less than 2°, which shows that in a uniform linear array, the CS-DOA-DBSCAN algorithm has better performance when the number of arrays is larger than the number of sources. When the number of signal sources is close to the number of array elements, the estimation results are prone to large deviations.

### 4.3. Performance Analysis of DOA Experiment at Close Angles

**Figure 11:**
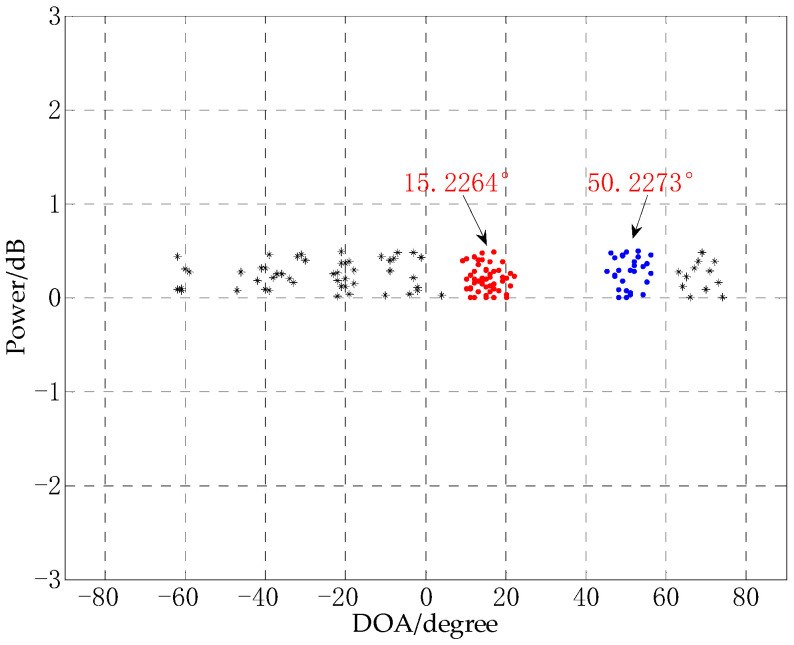
DOA estimation experiment of close angles.

The resolution of the DOA estimation algorithm is also one of the important indicators for testing the performance of the algorithm. In this experiment, the experimental performance of the CS-DOA-DBSCAN algorithm in the presence of close angles are tested. The experiment uses an 8-element uniform linear array, the array element spacing is 0.5 m, the number of snapshots is 10, the SNR is −10 dB, and the three signal sources are 15°, 18°, and 50°. The experimental results are shown in Figure 11.

It can be seen from Figure 11 that in a low SNR environment, the two close angles 15° and 18° are not correctly estimated by the CS-DOA-DBSCAN algorithm. Instead, the two angles are clustered into one angle. According to the principle of DBSCAN, when there are some noise points between close angles, and these noise points are located in the neighborhood of the close angles, wrong results may occur. In a low SNR environment, it is a challenging task to achieve DOA estimation of close angles.

### 4.4. Performance Analysis of DOA Experiments under Different Snapshots

**Figure 12:**
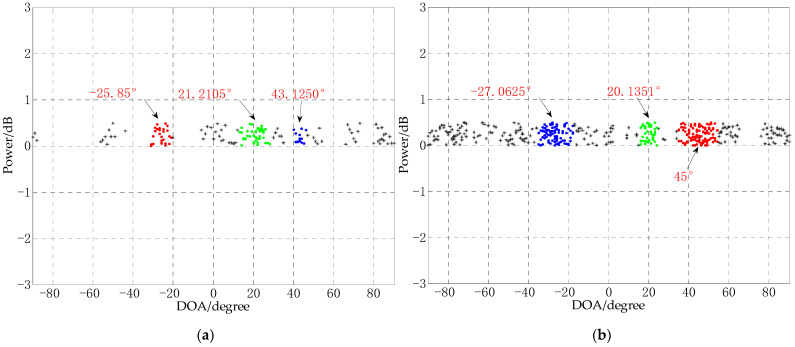
DOA estimation experiment under different number of snapshots, (**a**) snapshots = 10; (**b**) snapshots = 30.

In order to test the experimental performance of the CS-DOA-DBSCAN algorithm under different snapshots, in this experiment, 10 and 30 snapshots data are selected for the DOA estimation experiment. The other experimental conditions are the same, in which the SNR is −10 dB, the number of array elements is 8, the distance between the array elements is 0.5 m, and the three signal sources are −25°, 20°, and 45°. The experimental results are shown in Figure 12.

It can be seen from Figure 12 that in the DOA estimation experiment with the number of snapshots of 10 and 30, the CS-DOA-DBSCAN algorithm has obtained effective DOA estimation, but with the increase of snapshots data, the performance of the algorithm has not improved. It can be seen from Figure 12b that when the snapshots increases, the points near the real signal sources also increase greatly, and at the same time, the noise becomes denser. This will make the DBSCAN algorithm unable to distinguish the real signal sources from the noise points, leading to DOA estimation error. In addition, a large number of snapshots will cause a serious decrease in the computational efficiency of the algorithm. In this experiment, the experiment with 10 snapshots took 4.3538 s, and the experiment with 30 snapshots took 10.9101 s.

### 4.5. Performance Analysis of DOA Experiment of Non-Uniform Linear Array

**Figure 13:**
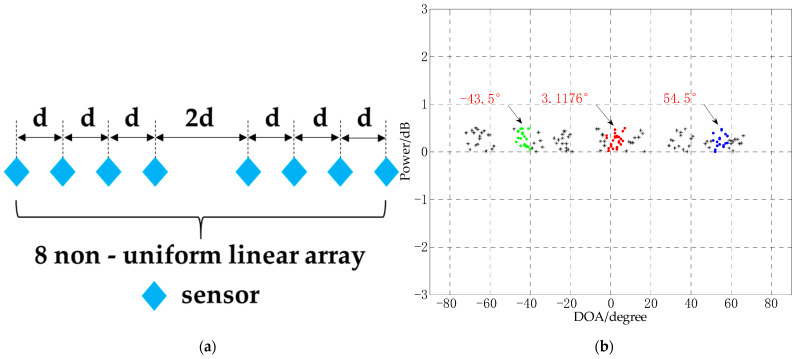
Non-uniform linear array DOA estimation experiment, (**a**) array model; (**b**) DOA experiment.

The non-uniform linear array has also received extensive attention from scientific researchers due to its flexible layout [40]. In this section, a nonlinear array experiment is designed to test the DOA experimental performance of the CS-DOA-DBSCAN algorithm. The number of array elements is 8, and the distance between array elements is shown in Figure 13a, d=0.5 m, the number of snapshots is 10, the SNR is −10 dB. In order to analyze the relationship between the performance of the CS-DOA-DBSCAN algorithm and the signal frequency and the sensor spacing at the same time, the 3 signal sources in this experiment are −45°, 5°, 55°, and their corresponding signal frequencies are 2000 Hz, 1500 Hz, 1000 Hz, respectively, The experimental results are shown in Figure 13b.

It can be seen from Figure 13 that the CS-DOA-DBSCAN algorithm can obtain effective DOA estimation in the experiment of non-uniform linear array, and the errors of the three DOAs are all less than 2°. Compared with the uniform linear array, the performance of the algorithm is not affected. In addition, the experimental results also show that CS-DOA-DBSCAN algorithm performance are not affected by the signal frequency and distance between sensors. This is because the algorithm uses the sparsity of the signal source in the spatial structure to estimate the DOA, and the signal frequency does not affect the sparsity of the signal source in the space, so the algorithm can also be used to process coherent signals. The following content will test the performance of the algorithm in the coherent signal DOA experiment.

### 4.6. DOA Estimation of Coherent Signals

**Figure 14:**
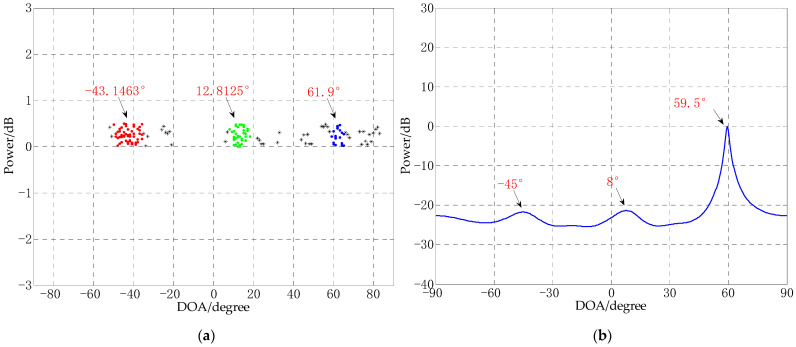
DOA estimation experiment for coherent signal. (**a**) CS-DOA-DBSCAN, (**b**) MUSIC.

In the real environment, due to the existence of multipath effects, the signal will inevitably appear coherent in the process of propagation, which will cause huge interference for the estimation of the DOA of the signal. In this experiment, the DOA estimation performance of the proposed algorithm in the case of coherent signals was tested, and compared with the MUSIC algorithm. The number of array elements is 8, the distance between array elements is 0.5 m, and the three incident angles are −45°, 10°, and 60°, respectively. The signals of −45° and 10° are coherent signals. The number of snapshots of the proposed algorithm is 10, and the SNR is −10 dB. In order to observe the influence of coherent signal on MUSIC algorithm, the snapshots of MUSIC algorithm are 1000 and the SNR is 0 dB. The experimental results are shown in Figure 14.

As can be seen from Figure 14a, when there are coherent signals, CS-DOA-DBSCAN algorithm can still get effective estimation under the conditions of small snapshots and low SNR. Because the DOA estimation principle based on CS only uses the sparsity of the signal source in space to estimate the DOA, which is not affected by the signal frequency. The coherent signals will not have significant impact on the DOA estimation results of CS-DOA-DBSCAN algorithm. It can be seen from Figure 14b that the spectral peaks of coherent signals are lost in the DOA estimation process of MUSIC. The reason is that the coherent signals lead to the loss of ranks when solving the covariance matrix of signal sources, which causes the failure of such methods in DOA estimation for coherent signals.

### 4.7. Comparison of Estimation Error of Different Algorithms under Different SNR

**Figure 15:**
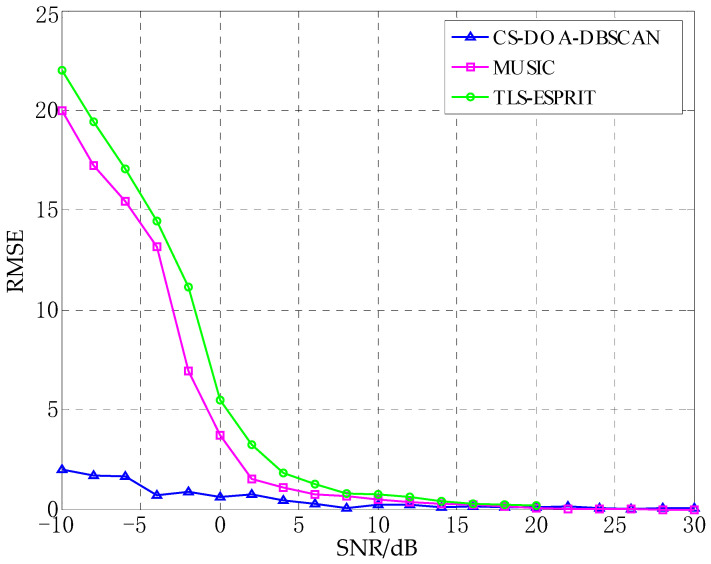
The RMSE of the DOA estimation of each algorithm under different SNRs.

In order to test the experimental performance of CS-DOA-DBSCAN algorithm under different SNRs with limited snapshots conditions. In this experiment, CS-DOA-DBSCAN, MUSIC, TLS-ESPRIT were used to estimate the DOA of signals in small snapshots for different SNRs. The average of the root mean square error (RMSE) of 50 experiments under each SNR was taken as the final results. Figure 15 shows the RMSE of the DOA estimation of each algorithm under different SNRs, which was from −10 dB to 30 dB.

It can be seen from Figure 15 that the RMSEs of the CS-DOA-DBSCAN algorithm are the smallest in the environment of small snapshots and low SNRs, their values are less than 2. Under the same environment, the DOA estimates obtained by MUSIC and TLS-ESPRIT have large errors. This shows that under the conditions of small snapshots and low SNRs, CS-DOA-DBSCAN algorithm has more advantages.

## 5. MEMS Vector Hydrophone Signal Processing

To verify the application effect of the proposed algorithm in practical engineering, the CS-DOA-DBSCAN algorithm is applied to the underwater acoustic direction finding of MEMS vector hydrophone in this section.

### 5.1. Experimental Equipment and Experimental Environments

**Figure 16:**
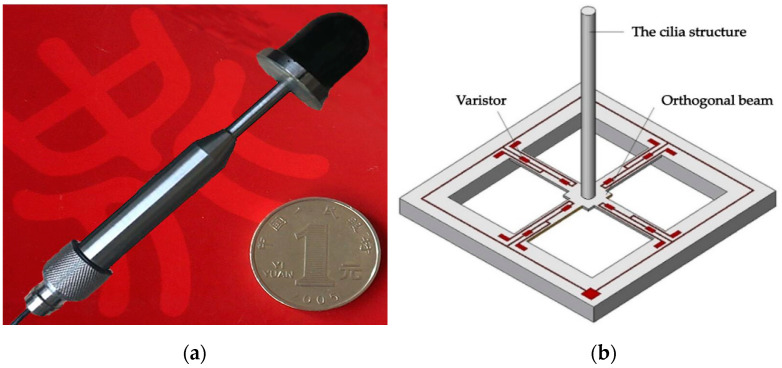
(**a**) MEMS vector hydrophone and (**b**) Cross-beam structure.

**Figure 17:**
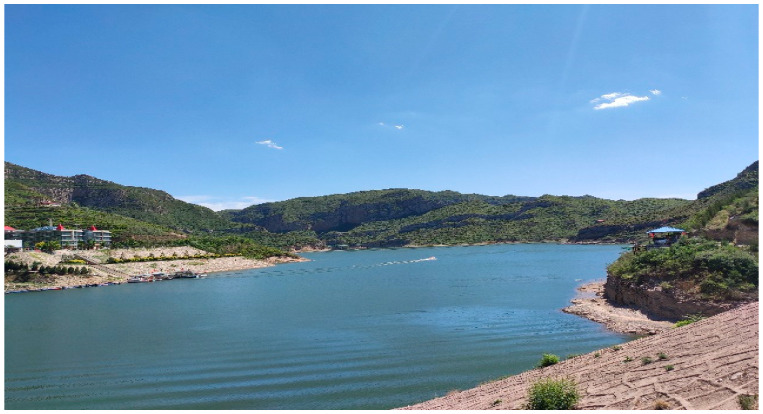
Reservoir environment.

The MEMS vector hydrophone used in this experiment is the ciliated bionic vector hydrophone developed by North University of China. The cilia in the center of the cross-beam structure of the hydrophone can perceive the water wave changes caused by the weak water sound, and convert the water sound signal into an electrical signal [41]. The experiment was carried out in a reservoir in Taiyuan, Shanxi. The reservoir has a wide water area. The average water depth of the area where the experiment is located is about 30 m, and the barrier-free water area exceeds 1 km. The cross beam structure of the MEMS vector hydrophone and the reservoir environment are shown in Figure 16 and Figure 17, respectively.

### 5.2. Lake Experiments

#### 5.2.1. Direction Finding Experiments for Single Source Underwater Acoustic Signal

**Figure 18:**
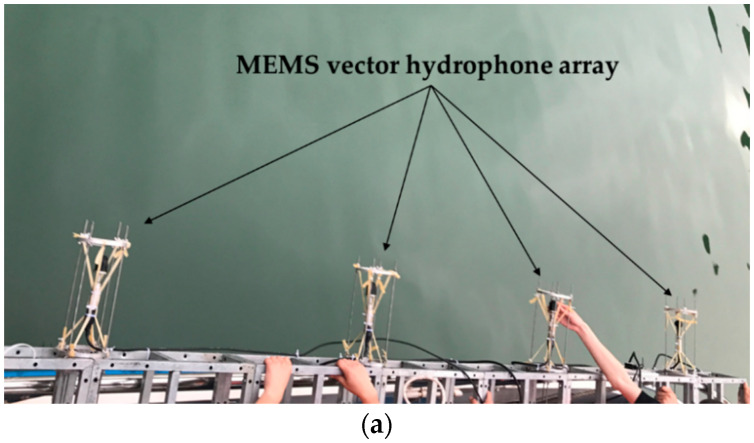

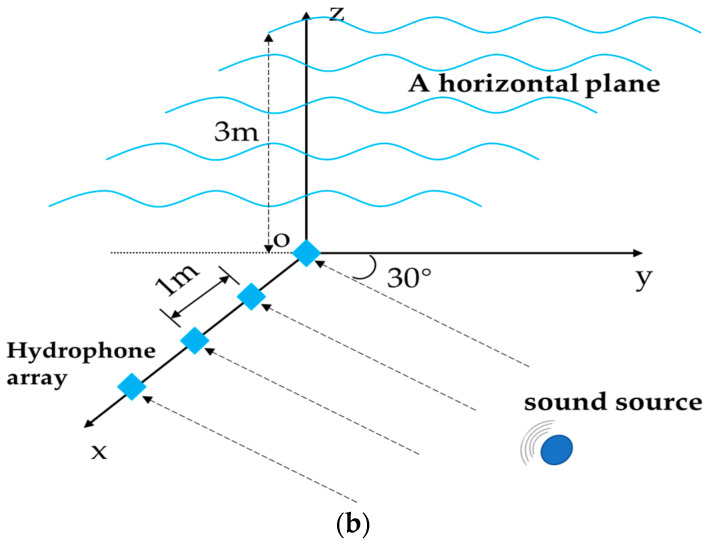
(**a**) MEMS vector hydrophone array and (**b**) schematic of the experiment.

In this experiment, the fish lip emission transducer is used for continuous sound generation, and the 4-element horizontal uniform linear array is used for underwater acoustic signal acquisition. Both the fish lip emission transducer and the horizontal linear array are placed at the depth of 3 m, and the sound source continuously emits the sound with a radio frequency of 315 Hz at 30° away from the horizontal uniform linear array. The distance between MEMS vector hydrophones is 1 m. Figure 18 shows the horizontal uniform linear array of 4-element MEMS vector hydrophone and the schematic diagram of the experiment.

**Figure 19:**
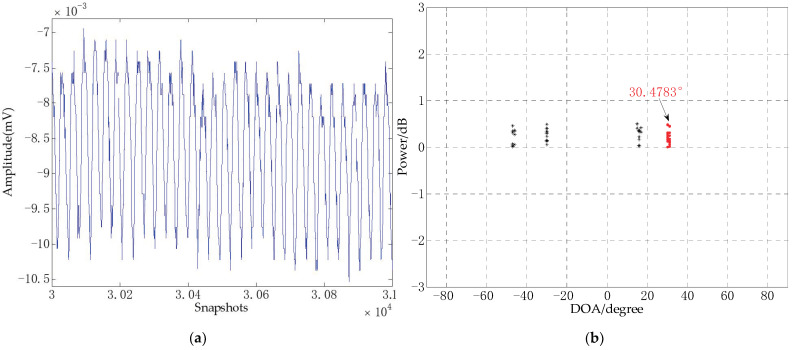
(**a**) The part of original signal and (**b**) DOA estimation.

First of all, because of the signals are collected by a four-element horizontal uniform linear array, multiple signals were generated. In this experiment, use the average value of the power spectrum entropy of the multiple signals to characterize the noise complexity of the signal source, and then the appropriate regularization parameters are selected. In this experiment, the regularization parameter is 12 and the number of snapshots is 30. Figure 19 shows the part of the original signal and the DOA estimation.

It can be seen from Figure 19a that there is glitch noise at the early peak of the original signal, and the waveform has some fluctuations. In Figure 19b, the angle error between the estimated result obtained by CS-DOA-DBSCAN algorithm under 30 snapshots and the real sound source is 0.4783°. It shows that CS-DOA-DBSCAN algorithm can get good DOA estimation in signal processing of MEMS vector hydrophone.

#### 5.2.2. DOA of Mixing Single Sound Source and Explosion Shock Wave

**Figure 20:**
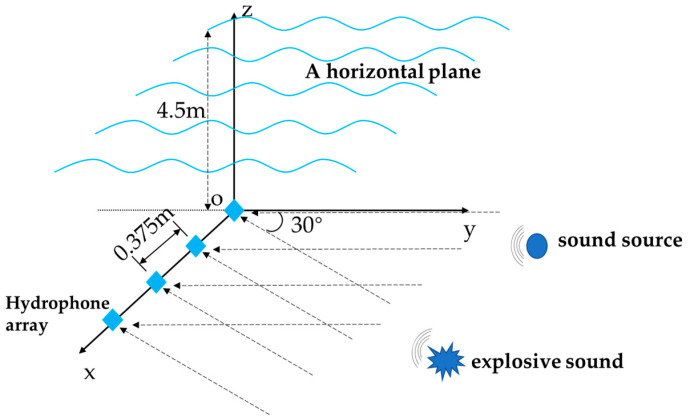
Schematic diagram of the mixing experiment of single sound source and explosion sound.

The real underwater sound environment is usually complex and changeable. The underwater sound may be reverberated or even coherent due to reflections during the propagation process. In order to test the performance of CS-DOA-DBSCAN algorithm in complex underwater acoustic environment, the following experiments are designed in this section. A horizontal uniform linear array of four elements are located 4.5 m below the horizontal plane to collect underwater acoustic signals, and a sound source continuously emits sound with a frequency of 2000 Hz in the vertical direction of the array. During the collection process of the array signal, an explosion sound was generated in the direction of 30° far away from the array. Figure 20 shows the schematic diagram of the experiment.

**Figure 21:**
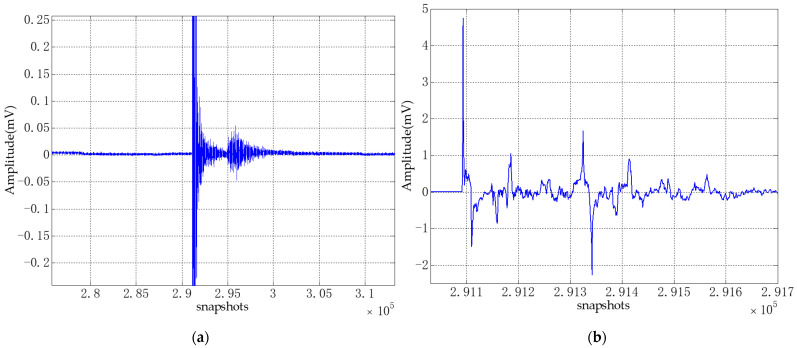
The part of original signal. (**a**) The signal near the shock wave, (**b**) Partial enlargement of the highest peak shock wave signal.

Because there was a brief explosion sound during this experiment, in order to visually see the effect of the explosion on the signal, part of the original signal collected by the hydrophone array is shown in Figure 21.

**Figure 22:**
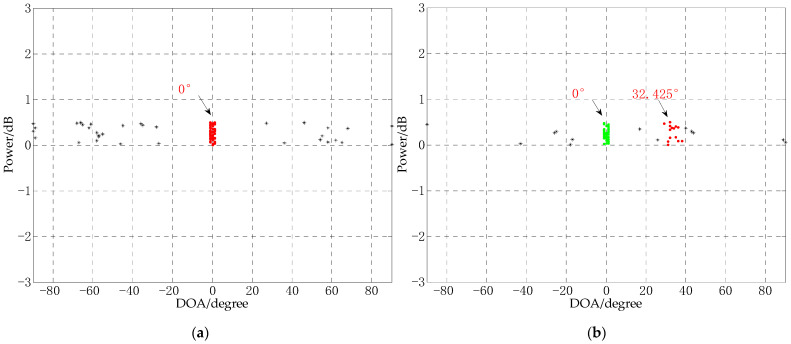
DOA estimation results. (**a**) DOA estimation of the stationary signal before the shock wave. (**b**) DOA estimation of the signal near the shock wave.

It can be seen from Figure 21a that the explosion sound produced a shock wave during the collection process of signal, and a larger disturbance was generated after the shock wave. It is because the explosion caused large fluctuations on the water body, and at the same time, it may be caused by the reflected sound waves of riverbank and riverbed. In Figure 21b, it can also be seen that the explosion sound has a large disturbance to the signal. In this experiment, 30 snapshots of the stable signal before the shock wave and 30 snapshots of the signal near the shock wave are selected for the DOA estimation experiment using CS-DOA-DBSCAN algorithm. According to the power spectrum entropy value of the signals, the regularization parameters were selected as 12 and 15, respectively, and the DOA estimation results are shown in Figure 22.

It can be seen from Figure 22a that there is only one sound source in the signal before the shock wave. CS-DOA-DBSCAN algorithm can obtain the correct DOA estimation in this period. It can be seen from Figure 22b that when the explosion sound occurs, the two incident signals of the continuous sound source and the explosion sound can be estimated at the same time using CS-DOA-DBSCAN. The DOAs are 0° and 32.425° respectively. Although there is some error between the estimated position of the explosion sound of 32.425° with the actual explosion position 30°, for short snapshots and DOA estimation in complex environments, CS-DOA-DBSCAN algorithm is still effective for DOA estimation. It is indicated that CS-DOA-DBSCAN algorithm can provide a new idea for MEMS vector hydrophone application in complex underwater acoustic environment.

## 6. Conclusions

Aiming at the DOA estimation problem under the conditions of small snapshots and low SNR, a method based on CS and DBSCAN was proposed in this paper. Simulation experiments and the application in signal processing of MEMS vector hydrophone verify the effectiveness of the proposed algorithm for DOA estimation in complex environments. The advantages and contributions of this algorithm are as follows:

(1) Regarding the selection of regularization parameters in the CS model, most of the literature selects the parameters based on experience, and there is no general judgment criterion. In this paper, the simulation results show that the adaptability of DOA estimation algorithm based on CS principle to noise is related to the selection of regularization parameters, and then the power spectrum entropy is proposed to represent the complexity of signal noise, which indirectly provides a reference for the selection of regularization parameters.
(2) Traditional DOA estimation algorithms usually need to predict the number of signal sources. In a low SNR environment, the performance of DOA estimation based on the CS principle will deteriorate, which is likely to cause misjudgment of the number of signal sources and DOA. The algorithm proposed in this paper can obtain the number of sources while obtaining effective DOA estimation, even under the condition of a small amount of abnormal snapshots.
(3) In the case of non-uniform linear array or signal coherence, the algorithm proposed in this paper can also obtain good DOA estimation results.

In addition, in a low SNR environment, the method in this paper has poor DOA estimation performance for close signals; the work will focus on the DOA estimation of close signal sources in a low SNR environment and its application in arrays of different structures.

## Data Availability

Not applicable.
